# Real-Time Two-Dimensional Mapping of Relative Local Surface Temperatures with a Thin-Film Sensor Array

**DOI:** 10.3390/s16070977

**Published:** 2016-06-25

**Authors:** Gang Li, Zhenhai Wang, Xinyu Mao, Yinghuang Zhang, Xiaoye Huo, Haixiao Liu, Shengyong Xu

**Affiliations:** Key Laboratory for the Physics & Chemistry of Nanodevices, and Department of Electronics, Peking University, Beijing 100871, China; pkloylee@pku.edu.cn (G.L.); wzh632@163.com (Z.W.); 15210624787@163.com (Y.Z.); huo_xiao_ye@163.com (X.H.); liutsunami@gmail.com (H.L.)

**Keywords:** temperature measurement, multiplexer, real-time, two-dimensional mapping, temperature sensor array

## Abstract

Dynamic mapping of an object’s local temperature distribution may offer valuable information for failure analysis, system control and improvement. In this letter we present a computerized measurement system which is equipped with a hybrid, low-noise mechanical-electrical multiplexer for real-time two-dimensional (2D) mapping of surface temperatures. We demonstrate the performance of the system on a device embedded with 32 pieces of built-in Cr-Pt thin-film thermocouples arranged in a 4 × 8 matrix. The system can display a continuous 2D mapping movie of relative temperatures with a time interval around 1 s. This technique may find applications in a variety of practical devices and systems.

## 1. Introduction

In the past decade, with the rapid development of smart cellphones, wearable and flexible electronics, bioscience and lab-on-a-chip systems, thermal management became a new technological frontier. For novel electronic devices and systems, besides focusing on chip-level thermal management alone, system-level solutions have received much attention. A real-time two dimensional (2D) mapping of the temperature distribution in individual chips and of the whole system could offer unique information for failure analysis, for the improvement of devices and systems, and even for finding optimized dynamic solutions for thermal management [1,2]. For biosciences research, a real-time 2D mapping of temperatures in individual live cells may reveal many unknown phenomena at the single cell and sub-cell levels. For micro- and nano-fluidic systems, real-time 2D mapping of temperature distribution is helpful for a better understanding of local bio-chemical reactions and for better control of the devices. Many techniques have been developed for real-time mapping of surface temperatures, such as infrared imaging, liquid crystal thermography, fluorescence imaging and scanning thermal microscope (SThM), etc. [3,4,5,6,7]. By analyzing the tiny density changes in 80-nanometer-thick aluminum wires using the electron energy loss spectroscopy technique, Mecklenburg et al. recently obtained 2D maps of temperature at the nanoscale in a scanning transmission electron microscope [8]. The temperature data in contactless measurements with infrared or fluorescence imaging are obtained indirectly by calculating shifts in the measured thermal excitation spectra, which usually leads to poor spatial and temporal resolution. The SThM technique measures the temperature by scanning the target surface with a tiny thin film thermocouple or thermal resistance mounted on its probe tip, therefore it has a superior spatial resolution, but the short contact time in the scanning process results in large temperature errors. Due to the requirement for a complicated measurement system, these methods are not applicable for moving objects, or for systems placed under a sealing cover. In these cases, built-in thermal sensors such as resistance temperature detectors (RTD) [9,10], diode sensors [11], and thin-film thermocouples (TFTCs) [12] are better candidates. As passive sensors, TFTCs provide stable performance with a short dynamic response time of 10^−9^–10^−6^ s [13,14,15], unique flexibility in size, and materials [12,16,17,18,19]. In several kinds of TFTCs a moderate thermal power (S) up to 19–26 μV/K and a high temporal resolution of 1–10 mK have been realized [12,17,19]. It was also demonstrated that TFTC arrays could be used to measure high temperatures and give the same readings as commercial K-type thermocouples [20]. A TFTC with a total sensor width of 140 nm, fabricated in a three dimensional (3D) stacking structure, was reported recently, which opens a door towards 2D mapping of local temperatures at the nanoscale with built-in TFTCs [21].

For displaying the 2D distribution of local temperatures T at a chosen time *t_0_*, one needs data T(*x*,*y*,*t_0_*), where (*x*,*y*) is the location coordinates. This can be achieved by traceback after obtaining the complete set of time-resolved data [12]. However, to show a real-time 2D mapping of local temperatures, one needs to obtain data T(*x*,*y*,*t*) and display the map quickly. This is challenging, as the number of measurement instruments is usually much less than the number of sensors. A low-noise multiplexer is therefore a proper solution. In this communication, we report a temperature measurement system with one nanovoltmeter, a hybrid electrical-mechanical multiplexer and an array of built-in Cr-Pt TFTCs arranged in an n × m matrix. We performed a dynamic 2D mapping of local temperatures on a sample surface under one or two heating sources. The minimum delay was around 1.0 s, depending on the number of sensors in the array, the rate of temperature change on the sample surface and the pixel density of each frame.

## 2. Materials and Methods

**Figure 1:**
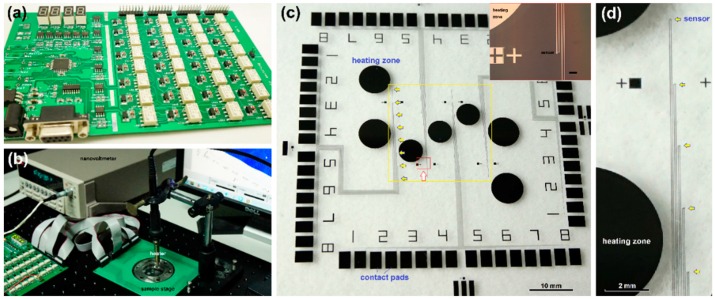
Optical photographs. (**a**) The multiplexer; (**b**) The testing system in a working state; (**c**) A sample with 32 Cr-Pt TFTCs arranged in a 4 × 8 matrix (in the yellow square region). Eight TFTC sensor junctions in the first row are highlighted with yellow arrows. The inset is an enlarged optical microscopy image of one TFTC junction region in the second row marked with a red frame, and the scale bar is 200 μm; (**d**) An enlarged view of the first row shown in (**c**).

The results reported in this work were obtained with homemade multiplexer circuits, which had an adjustable channel number from 10 to 100. Figure 1a is a photograph of a 32-channel multiplexer. The key elements for these multiplexers were solid relays (G6K-2F-Y-4.5V, Omron, Beijing, China). The “on” or “off” state of each relay was individually controlled with an electronic circuit, and the circuit and nanovoltmeter (2182A, Keithley, Beaverton, OR, USA) were controlled with an automation data acquisition system using the Labview program. This hybrid electrical-mechanical multiplexer reduced system noise to 1–2 μV in fast dynamic measurement process. By averaging ten continuous data points for the same sensor in the same state, the measurement error could be reduced to 0.2–0.5 μV. During measurement, each sensor was individually connected to the nanovoltmeter through a relay. For an array of sensors arranged in an n × m matrix, the nanovoltmeter measured them in sequence from sensor (1,1) to sensor (n,m), then started over again for the next run. The relay had a response time of 3 ms. To ensure a stable and reliable reading for the nanovoltmeter, a delay time of 5–20 ms after sending the triggering signal to each relay could be set in the operation menu, and another waiting time of 20 ms was set before the reading action. Therefore, for a 32-sensor array, the minimum time for each run of measurement was 0.8–1.28 s. For a 10 × 10 matrix, it took 2.5–4.0 s. If averaging of the readings for each sensor was necessary, each run of measurement would take longer.

Two display modes could be chosen. In a simple mode, the data for each run of n × m measurements were directly displayed in one frame, and for the locations between the sensor locations the data were calculated with a smooth interpolation algorithm. This is suitable for a slow dynamic process where the temperature varies smoothly and slowly within the total time needed for one run of measurement, 1 s. For a rapid dynamic process, we need to consider the time difference between sensor (1,1) and (n,m), i.e., ~1 s for a 4 × 8 array and ~3 s for a 10 × 10 array. This is just like the correction procedure used for taking digital photographs of a fast moving subject, we need to first fit the curves of temperature versus time for individual sensors, then obtain the temperature distribution at certain time T(*x*,*y*,*t_0_*) and display the 2D map. In this case more than two runs of data were needed and more calculations were performed to get T(*x*,*y*,*t*) following a procedure reported previously [12]. This leads to a much longer delay time of around 10–30 s, depending on the size of the sensor matrix, for the first frame of time resolved 2D map of the sample temperatures. Similarly, the interval between two neighboring frames also increases by several fold.

Figure 1b is a photograph of the whole measurement system in a working state, where the sample with embedded Cr-Pt TFTCs was mounted on a green printed circuit board (highlighted), and a heater (highlighted) was mounted on top of the sensor array. The distance of the heater tip to the sensor array, and the motion of the heater tip were both controllable with an *X*-*Y*-*Z* positioner. The heating power was set at 45 W. In some runs, two heaters with different heating powers were applied to check the performance of the system.

The Cr-Pt TFTCs were fabricated on 4-inch diameter glass substrates (Jingji Optics, Beijing, China) using standard cleanroom processes including photolithography (MJB4, SUSS MicroTec, Garching, Germany), oxygen plasma treatment, magneto-sputtering thin-film deposition (PVD 75, Kurt J. Lesker, Jefferson Hills, PA, USA), and lift-off. The power of magnetron sputtering system was 120 W with base vacuum of 1 × 10^−6^ Torr, and pure Ar was used as the operation gas. The bottom Cr layer and top Pt layer were both 100 nm in thickness. The widths of the Cr and Pt stripes were both 20 μm. The nominal thermopower (ΔS*) of the sensor was defined as ΔS* = Δ*V*/Δ*T*, where Δ*T* was the calibrated temperature between the hot and cold ends of each sensor and Δ*V* was the measured output voltage of the sensor. The calibration method is introduced in the Supplementary Materials.

## 3. Results and Discussion

The circuit noise in continuous dynamic measurements was 1.0–2.0 μV, corresponding to a temperature resolution of 0.2 K. Figure 1c shows a sample with an array of 4 × 8 Cr-Pt TFTCs located in a 2 × 2 cm^2^ sensing area (highlighted with a yellow square) on a 4-inch glass substrate. Locations for the first row of 8 TFTCs were marked with yellow arrows. The 64 contact pads of the 32 sensors were directly patterned from the same Cr or Pt thin films. They were located at the four edges of the sample, each edge consisting 16 pads for eight sensors. The inset is an enlarged optical microscopy image of two sensors in the second row (scale bar 200 μm). Figure 1d is a close view of the five sensors marked with yellow arrows in the first row as shown in Figure 1c. Seven circular patterns of Pt thin-film were fabricated together with the Cr-Pt sensors on the sample surface, three with a diameter of 2.2 mm and four with a diameter of 2.9 mm. These circular pads served as a uniform heating zone in the heating experiments. The cold ends of TFTCs were thermally connected on a metal stage to ensure the temperature in the cold ends kept at room temperature.

**Figure 2:**
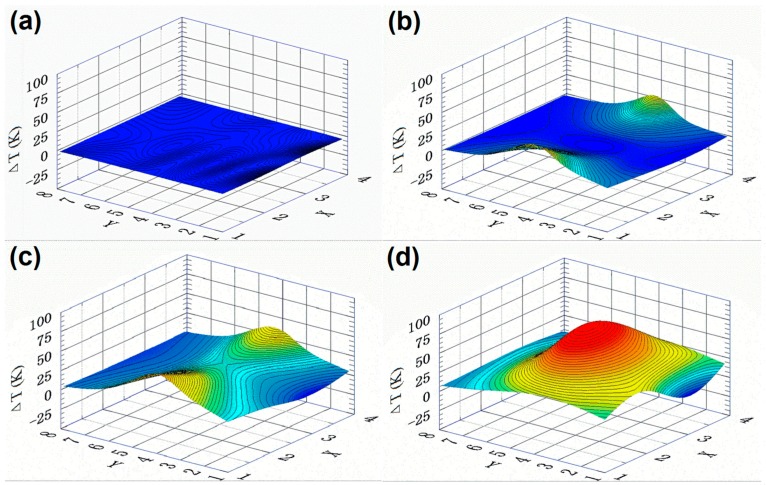
Measurement results taken from a continuous real-time mapping of a sample. (**a**) The resting state; (**b**) 20 s after two heaters are fixed at the two opposite edges at the same time; (**c**) After 50 s, these two edges increase in temperature; (**d**) After moving the two heaters both to the center of the sensing region and heating for 2 min, the central heating zone saturates in its temperature distribution.

The system worked well for both static and dynamic distribution of local surface temperatures in the sensing area. Figure 2 shows four typical results in a 3D manner, where the Δ*T* coordinate shows the temperature increase as compared to the cold ends, which are kept at room temperature. These four 3D maps are taken from a continuous movie of real-time mapping experiments. In the resting state, when no heating source is touching the sample surface, the measured temperature values of all the 32 sensors are near zero, as shown in Figure 2a. As shown in Figure 2b, two heaters are put on two opposite edges of the sensor area at the same time, and after 20 s, the two edges under the heating sources increase their temperatures by 28 K and 26 K at the peak positions, respectively. After heating for 50 s, these two peaks show increases of 37 K and 35 K, respectively, as shown in Figure 2c. Finally, when the two heaters are moved to the center region, it creates one larger heating zone and gradually the peak saturates at a temperature increase of 61 K, as shown in Figure 2d.

Note that the relative dynamic temperature distribution in the sensing area is most valuable, while the absolute temperature values shown in the Δ*T* coordinate are not accurate. This is because the Δ*T* value is calculated from the output voltage Δ*V* by Δ*T* = Δ*V*/ΔS*, but ΔS*, the nominal thermopower, is changed with the calibration method (see Supplementary Materials). For different systems, people may use different calibration methods to give reliable readings of the measured temperatures [12,22,23]. To reveal closely the tip temperature of the heater used in this work, ΔS* is chosen as 10 μV/K. This value was obtained by an on-site calibration procedure that the tip temperature was first determined with a small, standard K-type thermocouple and then the voltage output of each sensor was measured when the tip was put on the junction region, touching it with a thin polymer buffer layer (see Supplementary Materials).

**Figure 3:**
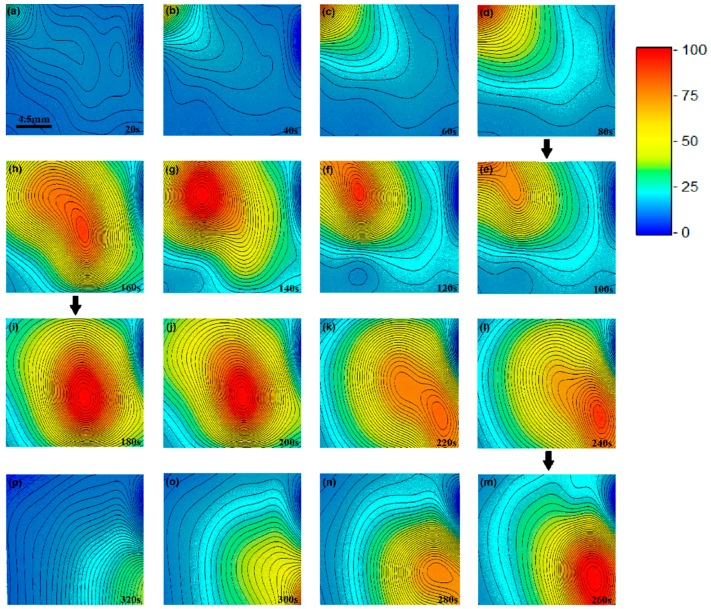
Experimental results taken from a continuous real-time mapping of 2D distribution of a sample. (**a**–**d**) Data for first 80 s, when a heater tip is moved from far away to the top-left corner, staying there for 80 s; (**e**–**p**) Data for the next 240 s, when the heater tip moves gradually from the top-left corner to the bottom-right corner. The movie of this process is available in the Supplementary Materials.

Figure 3 illustrates the real-time mapping of temperature distribution when a heat source moves from a location outside of the sensing region to the up-left corner, staying there for 80 s, then moved continuously across the sensing area from the top-left corner to the bottom-right corner. Figure 3a–d show the results of the first 80 s process, at *t* = 20, 40, 60 and 80 s: an increase in temperature by 90 K in the top-left. Figure 3e–p show the results from *t* = 100 s to *t* = 320 s, when the heater gradually moves to the central region, and to the bottom-right corner, and finally moves away from the sensing region. In this experiment, the moving heater tip touches the sample surface. But the tip is lifted up slightly when it is going to touch the sensors to avoid scratching damage. When the heating tip is lifted up shortly, the surface temperature decreases correspondingly, as shown in Figure 3e,h,k,n. The whole process was shown clearly in a video (see Supplementary Materials: Supplementary 1-Video of Figure 3).

## 4. Conclusions

We have presented here a temperature measurement system with a hybrid electrical-mechanical multiplexer and an array of TFTCs. By using this system, we have demonstrated dynamic 2D temperature mapping of a flat surface with 4 × 8 built-in Cr-Pt TFTCs. For slow dynamic processes, when the samples are under one or two moving heating sources, the minimum delay for the 2D frames of sample temperatures is 0.8–1.3 s. The delay and interval times depend mainly on the number of sensors used in the array and the pixel density required for each 2D frame. This technique is suitable for real-time 2D mapping of relative local temperatures of integrated circuit chips, flexible electronics, and microfluidics devices, where dynamic change of local temperature is not very fast. With improved software, it may also be applied in monitoring 3D temperature distributions of a complicated system.

## Supplementary Materials

The following are available online at http://www.mdpi.com/1424-8220/16/7/977/s1, Figure S1: An optical top-view photography of the hybrid electrical-mechanical multiplexer used in this work. (a) Connector to host PC; (b) Controller for relays; (c) Manual keys for reset of the controller; (d) LED Display; (e) Driver; (f) Relay matrix; (g) Connector to sensors; (h) Connector to nanovoltmeter, Figure S2: A schematic flow chart of the design and working mechanism whole measurement system. The dashed yellow frame indicates what are consisted in the multiplexer board, Figure S3: Typical calibration results of 8-cm long, Cr/Pt thin-film thermocouples, measured on a static calibration platform. The data are measured after the sample temperatures are stabilized at both hot and cold ends, which takes about 10 min for each data point. The fitting curve gives a nominal thermopower of 14.87 μV/K, Figure S4: Calibration results for one sensor in the sensing array, where the heater tip touches directly over the junction region with a thin buffer layer of dried super glue. Heat partially dissipates from the tip-touching point to the glass substrate. Linear fitting of the measurement data gives a nominal thermopower of 9.83 μV/K, Figure S5: Calibration results for one sensor in the sensing array, where in measurement the heater tip touches the substrate 300 microns away from the junction region. Due to strong dissipation of heat from the tip-touching point to the glass substrate, the linear fitting of the measurement data gives a nominal thermopower of only 4.99 μV/K, Video S1: Original video for Figure 3.
