# Supplementary material for: Real-Time Two-Dimensional Mapping of Relative Local Surface Temperatures with a Thin-Film Sensor Array
Source: Sensors (Basel). 2016 Jun 25;16(7):977. doi: 10.3390/s16070977 (PMC4969835; doi:10.3390/s16070977)
Supplement: Supplementary file 1 [file sensors-16-00977-s001.zip › sensors-16-00977-supplementary.pdf]

Supplementary Materials: Real-Time Two-Dimensional Mapping of Relative Local Surface Temperatures with a Thin-Film Sensor Array

Gang Li, Zhenhai Wang, Xinyu Mao, Yinghuang Zhang, XiaoyeHuo, Haixiao Liu, and Shengyong Xu

1. Lithographic Steps and Film Deposition Process of the Cr and Pt Layers

The Cr–Pt TFTC arrays were fabricated on 4-in glass by standard techniques of photolithography, thin film deposition, and lift-off. A photoresist 5350 (ALLRESIST, Strausberg, Brandenburg, Germany) was spin-coated on the glass substrate with a rotate speed of 4000 rpm for 1 min, and then baked at 110 °C for 3 min. After cooling to room temperature, the sample was exposed under 365 nm UV lights for 2.5 s, with an exposure dosage of 200 mJ/cm^2. After the photolithography (MJB4, 365 nm, SUSS MicroTec, Garching, Germany) and development, a 100-nm-thick Cr film was first deposited on the patterned substrate with a magnetron sputtering system (PVD 75, Kurt J. Lesker, Jefferson Hills, PA, USA), in which Ar was used as the processing gas and the sputtering power was 120 W. After the lift-off and cleaning processes, the substrate was re-patterned by photolithography again for the Pt layer. The Pt film, 100 nm thick, was deposited with the same sputtering system operating at 120 W. Before each deposition process of Cr and Pt films, low-power oxygen plasma was applied for 5 min to remove residual photoresist.

2. Design of the Multiplexer and the Working Flow Chart of the Circuit

The "multiplexer" is the key element of the measurement system described in this Communication. The top-view photograph and schematic flow chart of the circuit board of the multiplexer are presented in Figures S1 and S2.

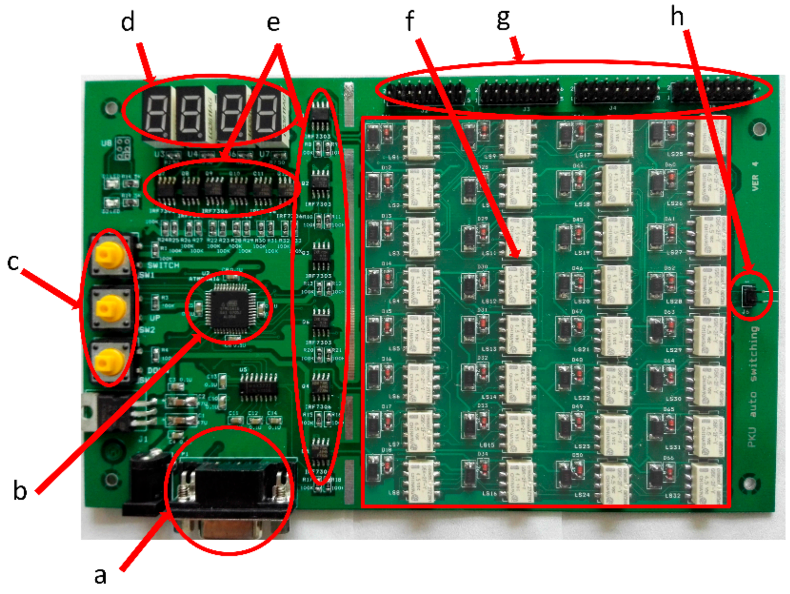

Figure S1. An optical top-view photography of the hybrid electrical-mechanical multiplexer used in this work. (**a**) Connector to host PC; (**b**) Controller for relays; (**c**) Manual keys for reset of the controller; (**d**) LED Display; (**e**) Driver; (**f**) Relay matrix; (**g**) Connector to sensors; (**h**) Connector to nanovoltmeter.

It consists an array of relays, whose number matches the number of temperature sensors used for a certain measurement. Each relay connects a sensor to the nanovoltmeter. Thanks to the mechanical connection when the relay is turned on (powered electromagnetically, controlled by the

"driver" chip shown in Figure S1), the system noises contributed by the on/off switching and the relay itself are as small as in the level of 0.1–1.0 micro-volt. This ensures a reliable reading of the sensor output voltage with the nanovoltmeter. The power supply for each relay is controlled by the "driver" chips of the multiplexer. The command for action of the "driver" is controlled by the "controller" chip shown in Figure S1, and the "controller" chip is programed by a Labview software running in the host PC. Several manual keys are used to reset the status of the "controller" chip. During measurement, the running status of each sensor is displayed with the LED lights (region "d" in Figure S1). The measured data are processed in real-time and displayed on the PC screen with a small delay of 1–10 s, depending on the number of sensors in the array, and the requirement of the 2D mapping quality.

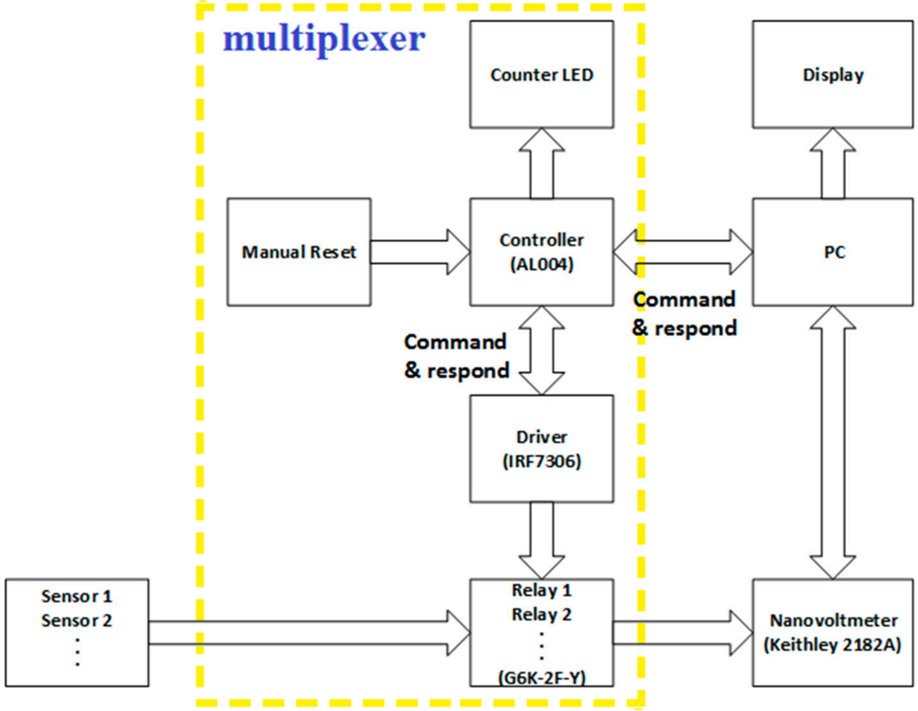

Figure S2. A schematic flow chart of the design and working mechanism whole measurement system. The dashed yellow frame indicates what are consisted in the multiplexer board.

3. Calibration of the Cr/Pt Thermocouple

Indeed the Cr/Pt thin-film thermocouples have been calibrated by three different ways. For the conventional way, we used two separate stages for the hot zone and cold zone. The hot zone was heated and maintained at a set temperature with a flat-plate heater. The cold zone was maintained at a set temperature by water cooling and an electrically cooling unit (working under the Peltier effect). Two commercial K-type thermocouples were used to measure the temperatures of the hot and cold zones, respectively.

Long Cr/Pt thin-film thermocouples, with stripe widths ranging from 10 microns to 100 microns and stripe length up to 8 cm, were made onto 4″ glass (or Si) wafers by standard cleanroom techniques as described in this paper. During calibration experiments, the wafer was mounted bridging the hot zone and cold zone, where the hot ends of the thermocouples were located on the hot zone and the cold ends of the samples were located on the cold zone. Meanwhile, a third commercial K-type thermocouples (TCs) was measured in parallel to the samples under test as a comparison, i.e., it indeed measured the temperature difference between the hot and cold zones.

Typical calibrated results were shown in Figure S3. The data were obtained in a static approach, i.e., when both of the hot and cold zone were kept at almost equilibrium states (with negligible temperature fluctuation), which took around 15 min. Here the linear fitting curve gives a

thermopower (sensitivity) of 14.87 μV/K. However, this thermopower changes slightly with film thickness and substrate; however, it is not sensitive to the stripe width. Cr/Pt thin-film thermocouples with varied film thickness and substrates all show a thermopower of 15.0±2.0 μV/K.

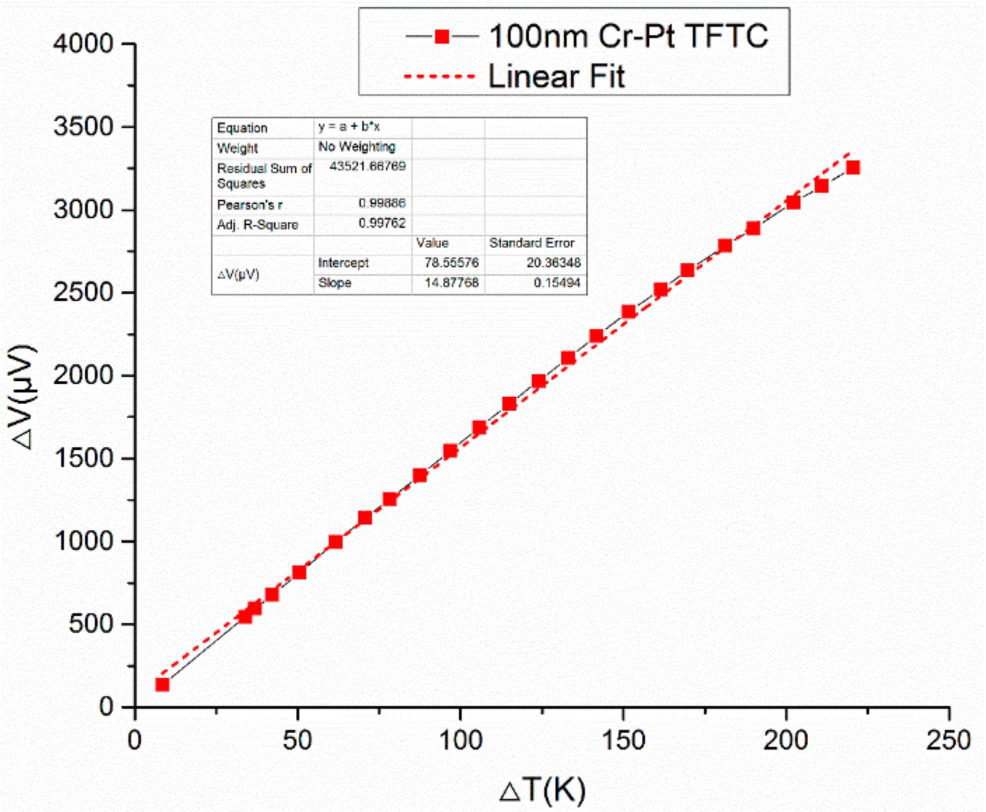

Figure S3. Typical calibration results of 8-cm long, Cr/Pt thin-film thermocouples, measured on a static calibration platform. The data are measured after the sample temperatures are stabilized at both hot and cold ends, which takes about 10 min for each data point. The fitting curve gives a nominal thermopower of 14.87 μV/K.

But in the present work for real-time 2D mapping experiments, the heating source was moving quickly during the measurement procedure. We controlled the tip position of a heater tip (set at certain heating power), and let it touching the surface of the substrate where our thermocouple array was fabricated, and moved it across the surface. When the tip was going to meet the sensor stripes, we lifted the tip a bit to avoid damage of the sensor, and then let it touching the substrate surface again when it passed the sensor.

In order to reveal closely the heater tip temperature, we performed the calibration in the following way. By directly touching the tip on a standard commercial K-type TC surface for 2 minutes, the reading of the TC was considered as the tip temperature. Then we let the heater tip touching directly the junction area of a Cr/Pt thin film TC on our sample. However, we had covered the junction region with a thin solid layer of super glue (Glue "502", Fuzelin Inc., Dongguan, Guangdong, China), which protected the junction area but also dissipated the heat transferred from the heater tip to the substrate, as the area of the glue layer was larger than the junction area. Figure S4 shows one of the measurement results. It gives a thermopower of 9.83 μV/K.

We also conducted other tests. Similarly, we first set the heater tip at certain temperature, and read the tip temperature with a commercial K-type TC by touching tip directly on the TC surface. Then we let the heater tip touching a point on the substrate, which was about 300 microns away from the junction region of each sensor. Then the temperature of the heater tip was increased to another set point and the procedure was repeated. Figure S5 presents a calibration result of one of the sensors. By this way we obtained a "nominal thermopower" of 4.99 μV/K.

Clearly, in the current heating manner used for this work, the real temperature of the hot zone was higher than what we measured through the Cr/Pt thin-film TC array. In Figures 2 and 3 of the paper, we had used a sensitivity of 10 μV/K for the transferring of measured output voltages directly into the increase of temperatures.

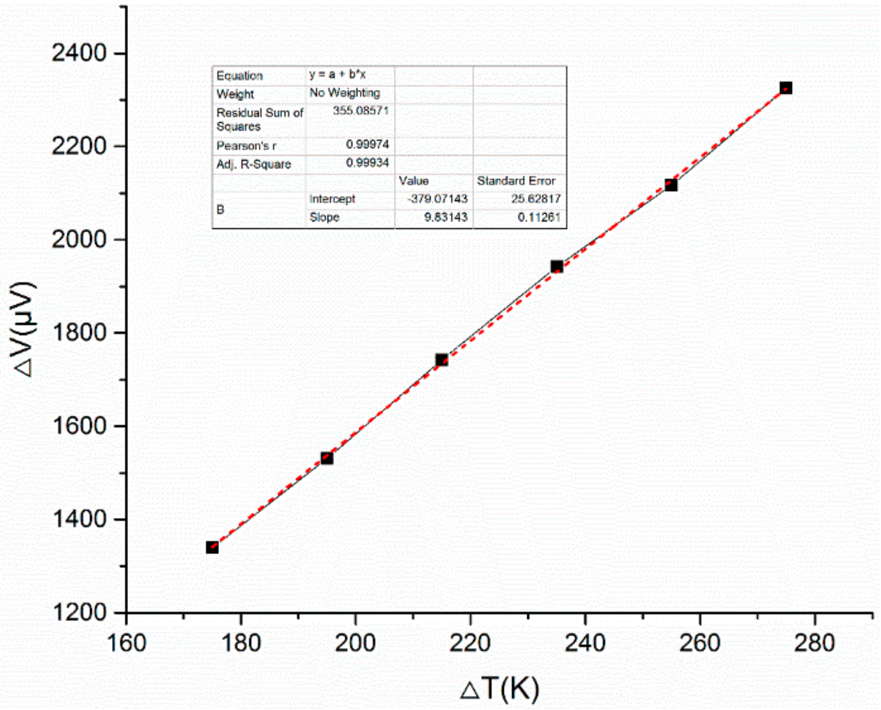

Figure S4. Calibration results for one sensor in the sensing array, where the heater tip touches directly over the junction region with a thin buffer layer of dried super glue. Heat partially dissipates from the tip-touching point to the glass substrate. Linear fitting of the measurement data gives a nominal thermopower of 9.83 μV/K.

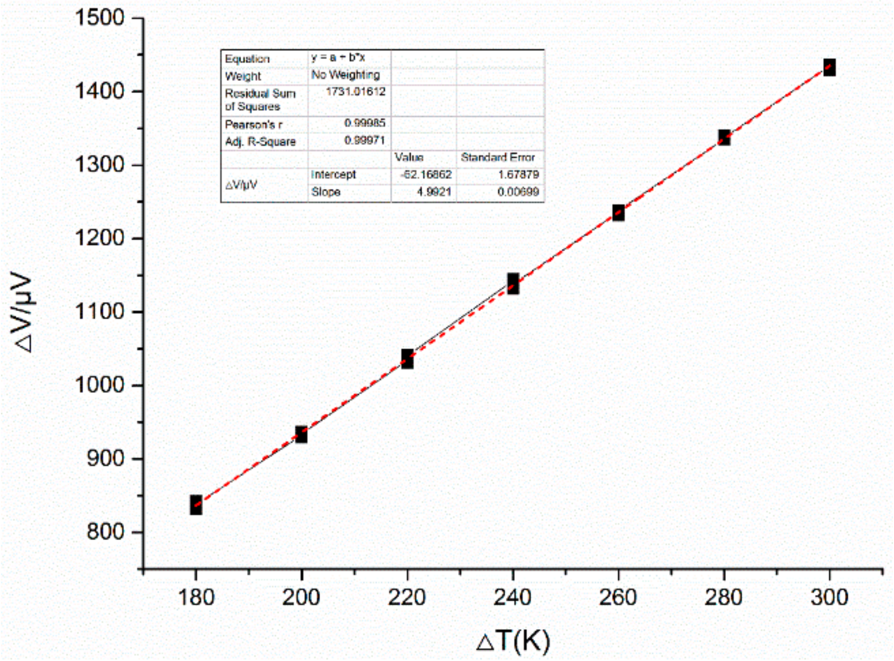

Figure S5. Calibration results for one sensor in the sensing array, where in measurement the heater tip touches the substrate 300 microns away from the junction region. Due to strong dissipation of heat from the tip-touching point to the glass substrate, the linear fitting of the measurement data gives a nominal thermopower of only 4.99 μV/K.
